# Anthelmintic Effect of* Bacillus thuringiensis* Strains against the Gill Fish Trematode* Centrocestus formosanus*

**DOI:** 10.1155/2016/8272407

**Published:** 2016-05-17

**Authors:** Luis Javier Mendoza-Estrada, Víctor Manuel Hernández-Velázquez, Iván Arenas-Sosa, Fernando Iván Flores-Pérez, Jorge Morales-Montor, Guadalupe Peña-Chora

**Affiliations:** ^1^Facultad de Ciencias Agropecuarias, Universidad Autónoma del Estado de Morelos, Avenida Universidad 1001, Colonia Chamilpa, 62209 Cuernavaca, MOR, Mexico; ^2^Centro de Investigación en Biotecnología, Universidad Autónoma del Estado de Morelos, Avenida Universidad 1001, Colonia Chamilpa, 62209 Cuernavaca, MOR, Mexico; ^3^Departamento de Medicina Molecular y Bioprocesos, Instituto de Biotecnología, Universidad Nacional Autónoma de México, Campus Morelos, Avenida Universidad 2001, 62210 Cuernavaca, MOR, Mexico; ^4^Departamento de Inmunología, Instituto de Investigaciones Biomédicas, Universidad Nacional Autónoma de México, AP 70228, 04510 México, DF, Mexico; ^5^Centro de Investigaciones Biológicas, Universidad Autónoma del Estado de Morelos, Avenida Universidad 1001, Colonia Chamilpa, 62209 Cuernavaca, MOR, Mexico

## Abstract

Parasitic agents, such as helminths, are the most important biotic factors affecting aquaculture, and the fluke* Centrocestus formosanus* is considered to be highly pathogenic in various fish species. There have been efforts to control this parasite with chemical helminthicides, but these efforts have had unsuccessful results. We evaluated the anthelmintic effect of 37 strains of* Bacillus thuringiensis* against* C. formosanus* metacercariae* in vitro* using two concentrations of total protein, and only six strains produced high mortality. The virulence (CL_50_) on matacercariae of three strains was obtained: the GP308, GP526, and ME1 strains exhibited a LC_50_ of 146.2 *μ*g/mL, 289.2 *μ*g/mL, and 1721.9 *μ*g/mL, respectively. Additionally, these six* B. thuringiensis* strains were evaluated against the cercariae of* C*.* formosanus*; the LC_50_ obtained from the GP526 strain with solubilized protein was 83.8 *μ*g/mL, and it could be considered as an alternative control of the metacercariae and cercariae of this parasite in the productivity systems of ornamental fishes.

## 1. Introduction

Among the most important factors affecting aquaculture are parasitic agents such as helminths, and one of the most important is the fluke* Centrocestus formosanus* [1] (Digenea: Heterophyidae), which has been found in a wide variety of fish species and is considered highly pathogenic in various fish species [2]. It spreads very quickly, has been recorded in several countries, and has medical importance because it can infect humans [3, 4]. In the municipality of Vientiane, the Lao People's Democratic Republic, a case of zoonosis was reported where seven patients were infected with* C*.* formosanus* [5]. There have been efforts to control this parasite using praziquantel with unsuccessful results [6] and with reports of resistance to anthelmintics [7–9]. Moreover, the use of large amounts of veterinary drugs in aquaculture can contribute to environmental stress [10]. Hence, it is necessary to implement effective measures for its prevention and control. The larval stages of several trematodes in many vertebrate hosts could be susceptible to new treatments of control [11]. Actually, the use of bacterial additives in modern aquaculture has demonstrated potential to improve the health of fish and reduce their mortality in culture by optimizing the water quality and reducing pathogen load [12]. Spores of Gram-positive bacteria of the genus* Bacillus* have advantages over vegetative cells because they remain stable for long periods of time, have the ability to grow rapidly, tolerate a wide range of physiological conditions, involve simple production processes, and are inexpensive. They can be used in commercial products because they are naturally suitable for ingestion by animals, have antagonistic effects on pathogenic microorganism, and are naturally ingested by animals [13]. The species* Bacillus thuringiensis* produces proteins that are toxic and highly specific against a variety of pests such as protozoa, insects, helminths, and mites of agricultural and veterinary importance [14], and they are harmless against nontarget insects and vertebrates. Thus, commercial bioinsecticides have been developed based on this species [15], which makes* B*.* thuringiensis* a suitable control alternative for* C*.* formosanus*.

## 2. Materials and Methods

### 2.1. Source of* Bacillus thuringiensis* Strains

The evaluated strains were provided by the* B*.* thuringiensis* collection from the Vegetal Parasitology Laboratory of the Center of Biological Research of the Autonomous University of the State of Morelos, Mexico. Three strains (IB-16, IB-61, and IB-62) were from the* B. thuringiensis* collection of the Institute of Biotechnology of the National Autonomous University of Mexico and have an anthelmintic effect on nematodes, and one strain was isolated from the metacercariae of* C*.* formosanus* collected in the field (Table 1).

### 2.2. Mass Production of Spore-Crystals

The strains were grown using solid medium Luria-Bertani (LB) and incubated at 28°C for 24 h to activate the bacteria. Then, they were seeded on a solid HCT medium, which contained (gL^−1^): Tryptone (Difco), 5; casamino acids (Difco) 2, KH_2_PO_4_ after sterilization, 3.4; MgSO_4_·7H_2_O, 0.012; MnSO_4_·4H_2_O, 0.003; ZnSO_4_·7H_2_O, 0.0028; Fe(SO_4_)_3_·7H_2_O, 0.02; CaCl_2_·2H_2_O, 0.147; and 10% glucose, were added [16]. The pH was adjusted to 7.0. Afterward, the strains were incubated for 72 h at 30°, and the protein crystals were observed in an optical phase-contrast microscope (Nikon eclipse 80i) at 100x. The spores and crystals produced by the* B. thuringiensis* strains were recovered using a bacteriological loop and suspended in 1 mL of sterile water. The spore-crystal complex was treated with 1 mM protease inhibitor PMSF (phenylmethylsulfonyl fluoride) to avoid protein degradation [17]. The total protein was quantified by the Bradford technique [18].

### 2.3. Source of* Centrocestus formosanus* Cercariae and Metacercariae

Infected snails (*Melanoides tuberculata*) were collected from the aquaculture production unit San Pedro Apatlaco located at 18°46′57.96′′NL and 98°57′59.87′′WL in the community of San Pedro Apatlaco in the municipality of Ayala, Morelos, Mexico. The snails were placed individually in 10 mL test tubes with pond water and exposed to artificial light (13 watt lamp) for two hours to stimulate the release of cercariae [19]. The snails were removed from the test tubes, and the cercariae were collected using a 200 *μ*L micropipette.

In the same place where the snails were present, we collected Japanese golden fish (*Carassius auratus*) with gill dysplasia, which is a sign of infection with* C. formosanus* metacercariae. The fish were 2-3 months old and had an average size of 30 mm. They were sacrificed following the protocols of the International Organization of Epizootics [20].* Centrocestus formosanus* metacercariae were obtained from the collected fish using a stereoscopic microscope (Motic SMZ-168) and micropins [21] to remove them carefully using a fine brush. Then, the cysts were placed in plates of 3 cm in diameter with a sterile 0.75% saline solution [22] and stored at 4°C until use in bioassays with the* B. thuringiensis* strains.

### 2.4. Viability of* Centrocestus formosanus* Cercariae and Metacercariae

The viability of the cercariae and metacercariae was verified with a stereoscopic microscope, and their motility was observed for 30 seconds (metacercariae within the cyst and cercariae in bottom of a Petri dish of 3 cm in diameter). Later, they were used in the respective bioassays [6, 23].

### 2.5. *In Vitro* Pathogenicity Bioassays

The experimental units used for the bioassays were 12-well cell culture plates (Corning Costar), and 30 newly emerged cercariae were placed per well. The determination of pathogenicity was performed using two concentrations of total protein (10 *μ*g/mL and 50 *μ*g/mL) and filtered pond water for the control group (3.0 *μ*m nitrocellulose membrane Millipore®). The commercial strain HD1, which produces proteins toxic to lepidopteran pests, was used as a negative control. Incubation lasted 6 h, and the experiments were performed in triplicate. The pathogenicity of the strains was measured by quantifying the number of dead metacercariae/cercariae, whereby motility was checked for 30 seconds using a stereoscopic microscope [6] and trypan blue stain [23]. The results were analyzed through an ANOVA and by Tukey's multiple mean comparison test (*P* ≤ 0.05).

### 2.6. Solubilization of* Bacillus thuringiensis* Protein Crystals

As the concentration of total protein was increased, different degrees of cloudiness were present which hindered the visualization of the toxic effects in cercariae; thus, we proceeded to solubilize the protein crystals to assess the anthelmintic effect to obtain the Lethal Concentration 50 (CL_50_) using the following protocols: sodium hydroxide solution (100 mM NaOH, 0.02%  *β*-mercaptoethanol, and 1 mM PMSF), 2 h at 4°C [24]; carbonate buffer (1 M Na_2_CO_3_ with a pH of 10.5, 0.02%  *β*-mercaptoethanol, and 1 mM PMSF, 2 h at 36°C [25]; 1 M Na_2_CO_3_ solution with a pH of 10.5, 80 mM NaCl, 0.02%  *β*-mercaptoethanol, and 1 mM PMSF), 2 h at 36°C [26]. Solubilized proteins were separated from insoluble material by centrifugation at 14 000 rpm for 20 min, and the supernatant was recovered and visualized by 10% SDS-PAGE. The proteins were dialyzed through membrane Spectra/Por*®2* (with a retention of 12.000 to 14.000 kDa molecules) for 12 hours in distilled water and stored at 4°C until use.

### 2.7. Lethal Concentration 50 (LC_50_)

We constructed a dose-response curve with solubilized proteins. We defined 10 concentrations based on the concentration which showed over 50% mortality on cercariae. Bioassays were performed in triplicate, and sterile water was used for the control group. The mortality was quantified at 24 hours by the techniques described above. The Lethal Concentration (LC_50_) was calculated using a Probit regression analysis with the Polo Plus statistical program [27].

## 3. Results

### 3.1. *In Vitro* Pathogenicity Bioassays

**Figure 1:**
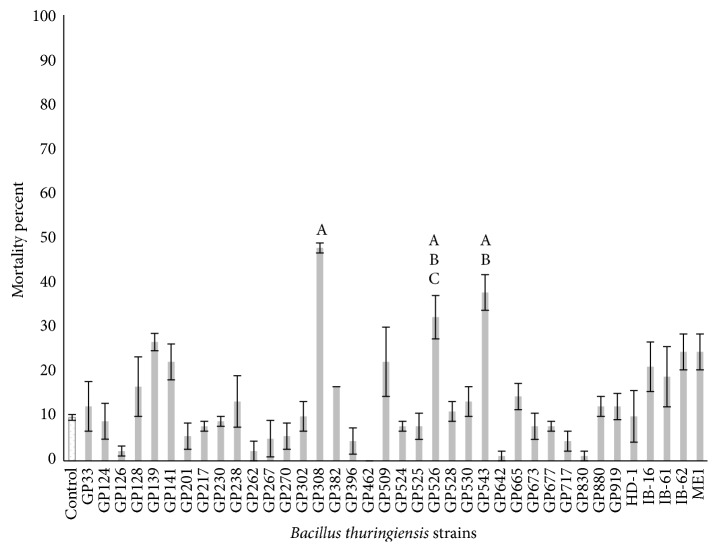
Metacercariae mortality rates after 6 hours of exposure to 10 *µ*g/mL spore-crystal mixtures. Bars with the same letter have no significant differences; error bars represent the $\overset{-}{X}$  ± SE (standard error).

**Figure 2:**
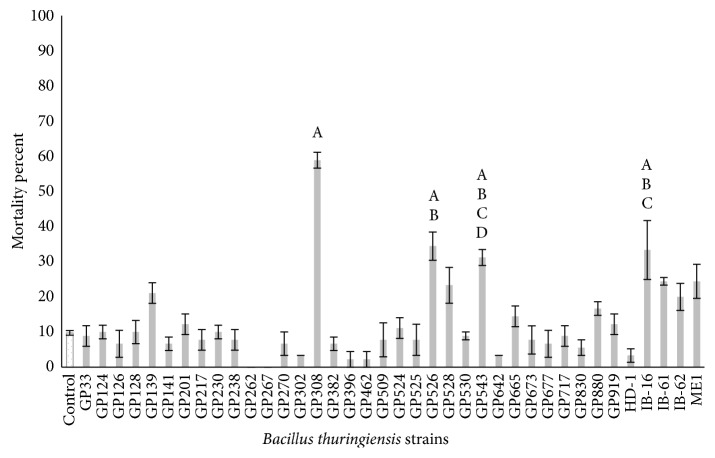
Metacercariae mortality rates after 6 hours of exposure to 50 *µ*g/mL spore-crystal mixtures. Bars with the same letter have no significant differences; error bars represent the $\overset{-}{X}$  ± SE (standard error).

**Figure 3:**
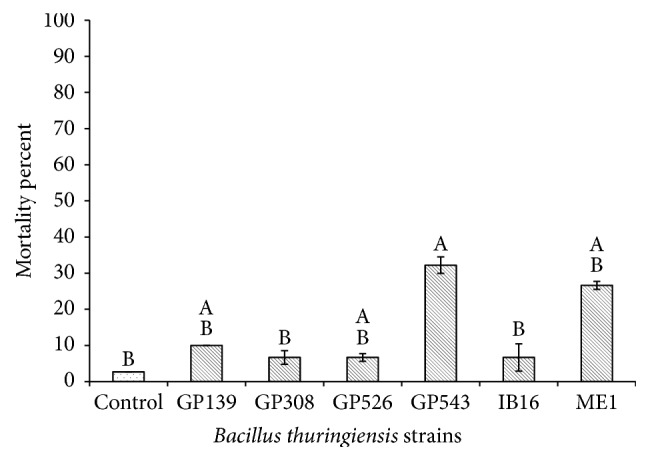
Cercariae mortality rates after 6 hours of exposure to 10 *µ*g/mL spore-crystal mixtures. Bars with the same letter have no significant differences; error bars represent the $\overset{-}{X}$  ± SE (standard error).

**Figure 4:**
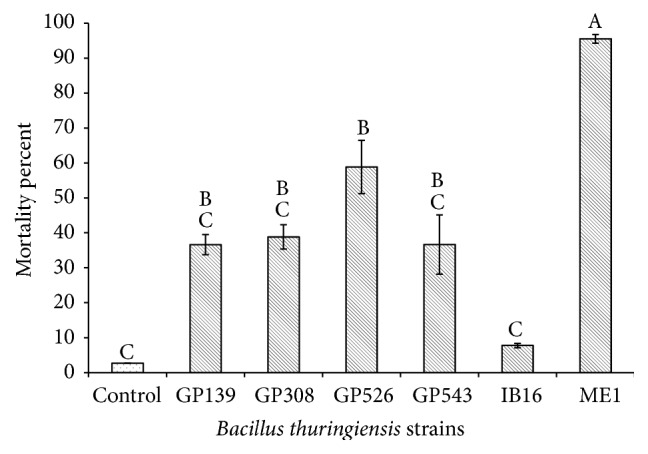
Cercariae mortality rates after 6 hours of exposure to 50 *µ*g/mL spore-crystal mixtures. Bars with the same letter have no significant differences; error bars represent the $\overset{-}{X}$  ± SE (standard error).

**Figure 5:**
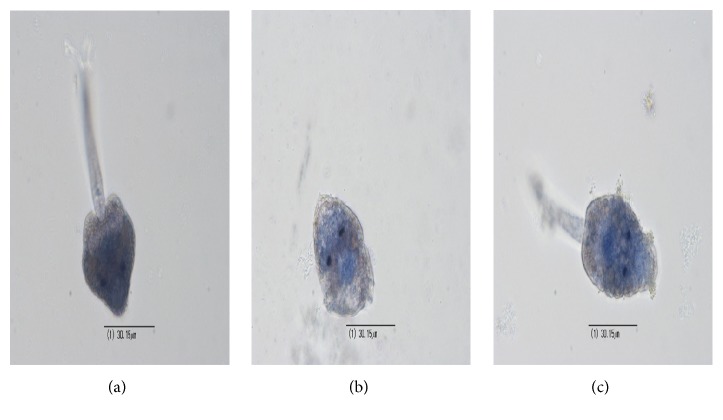
Lethal effects in* Centrocestus formosanus* cercariae: (a) control group (filtered pond water) 40x; (b) ME1 strain 50 *µ*g/mL total protein, cercariae stained with 0.4% blue trypan, 40x; (c) GP526 strain 50 *µ*g/mL total protein, 0.4% blue trypan, 40x. (Nikon DS-L1-5M).

**Figure 6:**
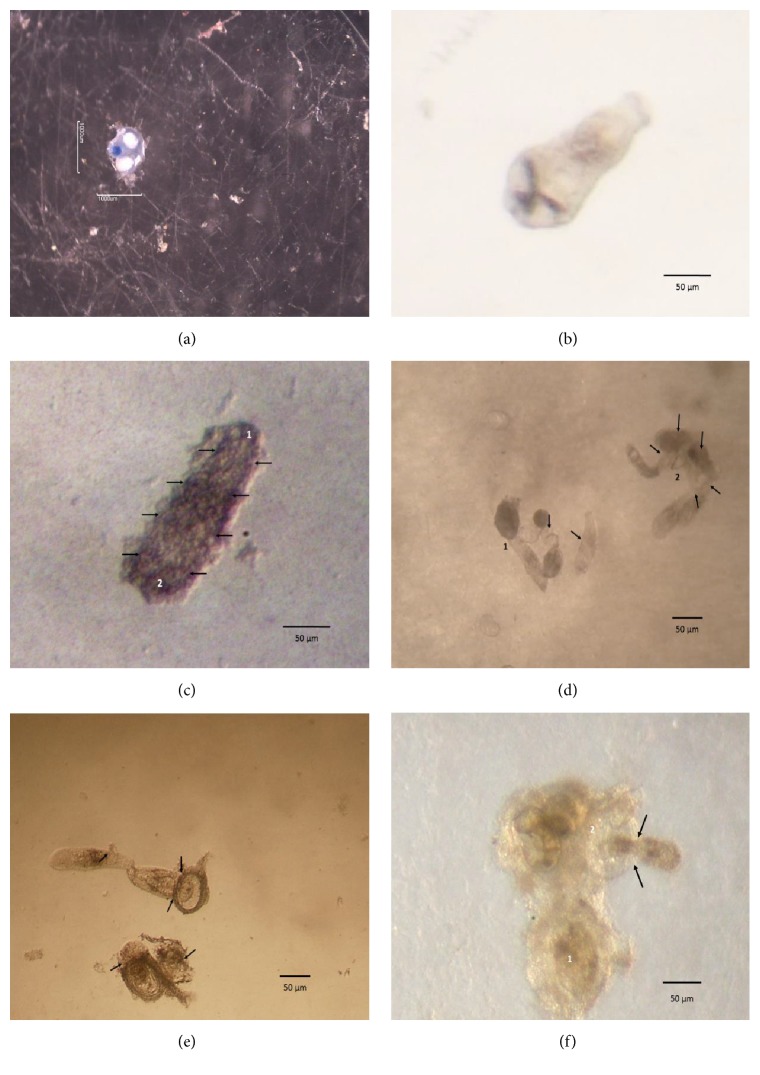
Lethal effects in* Centrocestus formosanus* metacercariae. (a) Control group (stained with blue trypan 0.4%, 2x Motic BA200); (b) control group, released metacercariae (10x Motic SMZ-168); (c) GP308 strain 50 *µ*g/mL total protein: (1) front and (2) back of released cyst metacercariae with detritus (arrows, 10x Motic SMZ-168); (d) GP526 strain 50 *µ*g/mL total protein: (1) encysted metacercariae and (2) metacercariae cyst released by the action of the total protein (arrows, 4x Motic SMZ-168); (e) IB-16 strain 50 *µ*g/mL total protein, metacercariae cyst released by the action of the total protein (arrows, 4x Motic SMZ-168); (f) ME1 strain 50 *µ*g/mL total protein: (1) encysted metacercariae and (2) metacercariae cyst released by the action of the total protein (arrows, 4x Motic SMZ-168).

For the bioassays with metacercariae, the strain GP308 at the 10 *μ*g/mL concentration exhibited the highest mortality of 47.8% followed by the strains GP543 (37.8%) and GP526 (32.2%); meanwhile, the remaining strains showed mortality rates lower than 30% (Figure 1), and there were significant differences between treatments (*P* ≤ 0.05). At a concentration of 50 *μ*g/mL the strain GP308 had the highest mortality rate (58.9%), followed by GP526 (34.4%) and IB-16 (33.3%); meanwhile, the other strains showed mortality rates lower than 30% (Figure 2), and there were significant differences between treatments (*P* ≤ 0.05). The strains GP139, GP308, GP526, GP543, IB-16, and ME1 were selected based on their percentage of mortality and statistical analyses compared with the other tested strains, and they were evaluated against* C. formosanus* cercariae. The GP543 strain at the 10 *μ*g/mL concentration showed a mortality of 32.2%; meanwhile, the other strains showed mortality rates lower than 10% (Figure 3), and there were no significant differences between treatments (*P* ≤ 0.05). At a concentration of 50 *μ*g/mL, the ME1 and GP526 strains showed the highest mortality rates of 92.5% and 58.8%, respectively (Figure 4), and by the Tukey test the ME1 strain was identified as the best treatment at this concentration. We also observed some structural alterations in the cercariae body. The majority of cercariae treated with GP526 total protein were characterized by a ballooning shape; they swam slowly in the bottom and then died. Some cercariae treated with the ME1 strain showed tail loss and tegument disruption which caused the release of their content to the external environment (Figure 5). In the case of metacercariae exposed to the spore-crystal complex of the strains, GP139, GP308, GP526, GP543, IB-16, and ME1 caused the release of the metacercariae cyst; in addition, the formation of debris around GP308 and IB-16 occurred (Figure 6).

### 3.2. Lethal Concentration 50

**Figure 7:**
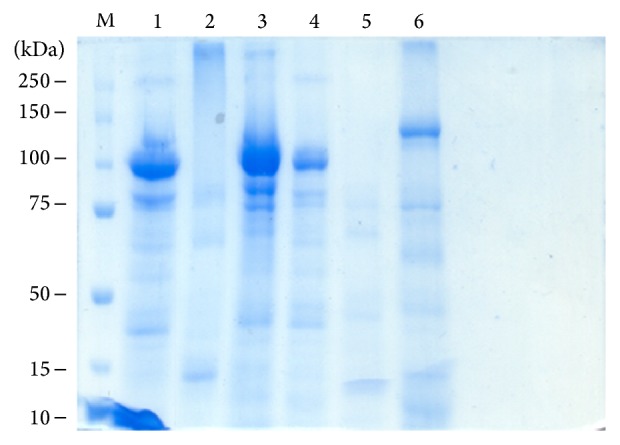
SDS-PAGE protein profile* Bacillus thuringiensis* strains toxic to* Centrocestus formosanus* cercariae. (M) Molecular weight marker* Precision Plus Protein All Blue Standards* (Bio-Rad): (1) GP139 strain, (2) GP308 strain, (3) GP526 strain, (4) GP543 strain, (5) IB-16 strain, and (6) ME1 strain.

Only three LC_50_ could be obtained with solubilized protein on metacercariae: GP308 = 146.2, GP526 = 289.2, and ME1 = 1721.9 *μ*g/mL. With the cercariae only one CL_50_ was obtained, and it was from the GP526 strain of 83.8 *μ*g/mL. The protein profile of the six candidate strains was obtained by SDS-PAGE (Figure 7). The IB-16 strain showed two major bands with a molecular weight of 70 and 20 kDa. The GP308 strain had four bands: 75, 70, 65, and 40 kDa; the ME1 strain had three bands: 150, 75, and 50 kDa; and for the GP139, GP526, and GP543 strains two bands were observed of 100 and 75 kDa.

## 4. Discussion

The use of chemical pesticides generates side effects on aquatic organisms and produces resistance in pathogenic bacteria and helminth parasites of fishes; thus, the use of beneficial bacterial aggregates has become a recurring practice in aquaculture due to the positive results that have been recorded, such as an improvement in water quality, the performance of water bodies, and decreased infectious agents of fishes and other aquatic organisms under cultivation [28, 29]. This study contributes to the knowledge on the use of bacteria as a potential anthelmintic agent, particularly the use of* B. thuringiensis* in the treatment of infections by parasite trematodes that affect aquaculture. There is evidence of* B. thuringiensis* anthelmintic activity, but it has been recorded primarily against nematodes of medical-veterinary importance [30, 31]. Thus, there are few reports of its effectiveness against trematode parasites. In this sense, there are few references to their use for the treatment of parasitic helminth infections in fish. The mortality observed in the trials of pathogenicity of* C. formosanus* cercariae was relatively higher than that reported in other studies that tested* in vitro* the recombinant protein Cry5B of* B. thuringiensis* used in the control of parasitic nematodes such as* Ancylostoma ceylanicum* [32],* Heligmosomoides bakeri* [33], and* Ascaris suum* [34]. In those studies different mortality rates were observed in adults, larval stages, and eggs, ranging between 50 and 95% using concentrations between 0.01 to 10 *μ*g/mL* in vitro* and 1 to 10 mg/Kg* in vivo* in murine models, with a decrease of activity at 48 hours on average. Unlike these works, however, in this research the mortality rate was recorded as 30–58% in* C. formosanus* encysted metacercariae and 60–95% in cercariae at a higher dose using 50 *μ*g/mL concentrations of the total protein of* B. thuringiensis*. For metacercariae, the cysts are structures of protection that guarantee the infection of their definitive host because they present histochemical characteristics that allow them to dissolve under the acidic conditions generated by the gastric juices of their definitive hosts [35]. The cyst formed by* C. formosanus* is reinforced by an outer layer formed by the host himself as immune system responses to infection, making the drug treatment very difficult [6]. We recorded the metacercariae cysts hatching as a result of the effect of the exposure to the* B*.* thuringiensis* proteins, and in some cases death of the metacercariae into the cyst was observed. Metacercariae were released into the medium after six hours of exposure to the protein, presenting changes in their morphology, such as the presence of small blisters on their integument (GP308 strain). The spore-crystal complex of some strains (IB-16, GP308, and ME1) caused the release of some metacercariae from the cyst, making them more susceptible to the proteins and environmental conditions. Some proteins [36] as well as lipids [37] have been identified within the histochemical structure of the cysts, and they are target molecules for Cyt toxins (membrane lipids [38] and virulence factors produced by* B. thuringiensis* such as cysteine protease (trypsin-like) [39]. Trypsin is commonly used to dissolve the cyst of the metacercariae to perform studies on the histochemistry and developmental biology of trematode [40]. Proteases produced by* B. thuringiensis* hydrolyze proteins that compose the cyst, enhancing the action of other toxins and virulence factors. Cyst rupture can facilitate the entry of bacteria and other microorganisms acting as metacercaria pathogens. The presence of the alkaline phosphatase molecule in the structure of adult flukes [35, 41] which makes them susceptible to the toxic activity of Cry toxins because they use this enzyme as a receptor [42] has also been reported.

It is known that the cercariae of flukes do not acquire any nutrients from their environment, move through with their energy reserves (mainly sources of glycogen) [43], and can survive for two to five days in water without infecting their host [44]. For this study, the cercariae were used for bioassays no more than six hours after they were collected to ensure that the mortality rates were due to the effect of the proteins rather than because they exhausted their energy reserves. The structural changes caused by the protein effect on cercariae and metacercariae are similar to those reported by Horák et al. [43]. Some cercariae had abnormalities in their morphology when they were treated with the GP526 and ME1 strains; they were characterized by a ballooning body shape (GP526 strain) and tegument disruption which caused the release of their content to the external environment (ME1 strain). This finding was similar to that reported by Horák et al. [43], where elutions of* B. thuringiensis israelensis* M-exotoxin were used; the authors mentioned that these structures are known as vacuolization and are due to separation from the body integument and are a clear symptom of poisoning by the exotoxin* B. thuringiensis israelensis* (leading effects of internal organs).

The strain GP526 was the most effective in the* in vitro* bioassays as it showed activity on the cercariae (infective stage) as well as the metacercariae (pathogenic parasite) of* C. formosanus*. This strain also exhibited a similar effect in another important helminth,* Dipylidium caninum* [23], presenting toxic activity on both adults and eggs. Trematodes as well as cestodes have a tegument [35], making them susceptible to the proteins produced by the GP526 strain.

The solubilization and/or purification of proteins from some strains of* B. thuringiensis* are more toxic in their anthelmintic effect [44, 45], a fact not observed with the proteins purified from the strain ME1. The best results were obtained using the complex spore-crystals in the pathogenicity tests with cercariae and metacercariae. This result supports the importance of the presence of spore-based formulations of* B. thuringiensis*. The results obtained in this work are promising, and the GP526 strain should be evaluated on fish farming where* C. formosanus* is a problem to determine whether it can control this parasite.

## Figures and Tables

**Table 1:** Origin of the *Bacillus thuringiensis* strains evaluated against *Centrocestus formosanus *cercariae.

Isolating source	Strains
Soil	IB-16, IB-61, and IB-62
*Dipylidium caninum* (Cestoda)	GP665
*Meloidogyne incognita* (Nematoda)	GP217
*Meloidogyne* sp. (Nematoda)	GP524, GP525, GP526, GP642, GP673, and GP677
*Rhipicephalus sanguineus* (Acarida)	GP141
*Rhipicephalus microplus* (Acarida)	GP543
*Varroa destructor* (Acarida)	GP509
*Psoroptes cuniculi* (Acarida)	GP880
*Periplaneta americana* (Blattodea)	GP33
Chrysomelidae (Coleoptera)	GP262
*Scyphophorus acupunctatus* (Coleoptera)	GP717
*Trialeurodes vaporariorum* (Hemiptera)	GP124, GP126, and GP302
Psyllidae (Hemiptera)	GP308
*Bemisia tabaci* (Hemiptera)	GP139 and GP830
*Triatoma* spp. (Hemiptera)	GP128 and GP201
*Phylloxera* sp. (Hemiptera)	GP230
*Rhopalosiphum maidis* (Hemiptera)	GP230 and GP238
Cicadellidae (Hemiptera)	GP270
Aphididae (Hemiptera)	GP382
*Myzus persicae* (Hemiptera)	GP919
Diaspididae (Hemiptera)	GP267 and GP396
Cercopidae (Hemiptera)	GP528
Lepidoptera	HD1
*Sphenarium* sp. (Orthoptera)	GP462
*Carassius auratus* (Pisces) infected gills with *Centrocestus formosanus* metacercariae	ME1
